# A Preliminary Italian Cross-Sectional Study on the Level of Digital Psychiatry Training, Knowledge, Beliefs and Experiences among Medical Students, Psychiatry Trainees and Professionals

**DOI:** 10.3390/healthcare10020390

**Published:** 2022-02-18

**Authors:** Laura Orsolini, Silvia Bellagamba, Virginia Marchetti, Giulia Menculini, Silvia Tempia Valenta, Virginio Salvi, Umberto Volpe

**Affiliations:** 1 Unit of Clinical Psychiatry, Department of Neurosciences/DIMSC, Polytechnic University of Marche, 60126 Ancona, Italy; l.orsolini@staff.univpm.it (L.O.); silvibella95@gmail.com (S.B.); silvia.tempia@gmail.com (S.T.V.); v.salvi@staff.univpm.it (V.S.); 2 School of Medicine and Surgery, Polytechnic University of Marche, 60126 Ancona, Italy; virginiamarchettivm@gmail.com; 3 Department of Psychiatry, University of Perugia, 06100 Perugia, Italy; giuliamenculini@gmail.com

**Keywords:** digital psychiatry, education, psychiatry training, telepsychiatry, trainees

## Abstract

The COVID-19 pandemic led to the implementation of digital psychiatry (DP), resulting in the need for a new skilled healthcare workforce. The purpose of this study was to investigate the level of training, knowledge, beliefs, and experiences of young mental health professionals and medical students in DP. An ad hoc cross-sectional survey was administered and descriptive analyses, Student’s *t* and ANOVA tests were conducted, together with an exploratory factor analysis, bivariate correlations and linear regression. Most of the sample (N = 239) declared that DP was never discussed within their academic training (89.1%), mainly revealing an overall lack of knowledge on the issue. Nevertheless, subjects mostly declared that DP represents a valuable therapeutic tool in mental health (80%) and that their training should include this topic (54.4%). Moreover, most subjects declared that digital interventions are less effective than face-to-face ones (73.2%), despite the emerging evidence that being trained in DP is significantly associated with the belief that digital and in-person interventions are comparable in their effectiveness (*p* ≤ 0.05). Strong positive correlations were found between the knowledge score (KS) and perceived significance index (PSI) (r = 0.148, *p* < 0.001), and KS and Digital Psychiatry Opinion (DPO) index (r = 0.193, *p* < 0.001). PSI scores statistically significantly predicted KS total scores (F(1, 237) = 5.283, R^2^ = 0.022, *p* = 0.022). KS scores statistically significantly predicted DPO total scores (F(1, 237) = 9.136, R^2^ = 0.037, *p* = 0.003). During the current pandemic, DP represented an ideal response to the forced physical distancing by ensuring the advantage of greater access to care. However, this kind of intervention is still uncommon, and mental health professionals still prove to be skeptical. The lack of formal training on DP during the academic years could be a limiting factor.

## 1. Introduction

The term telemedicine (TM) refers to a way of providing healthcare services through the use of innovative technologies, particularly Information and Communication Technologies (ICTs), in those circumstances in which the health professional and the patient are not in the same place. The term TM, literally meaning “healing at a distance”, was coined in 1970, referring to care programs addressed to geographically isolated patients [1]. The origins of this technology date back to the early 20th century, and in the subsequent decades rapidly evolved from the spread of the Erickson’s Bakelite telephones, the advent of the Internet and ICTs until the development of online video-communication services (e.g., Zoom Cloud Meeting, Google LLC Meet). This innovation has implemented increasingly effective remote health care, which nowadays represent a valid and cost-effective alternative to the traditional in-patient interventions in various specialty areas of medicine [2,3,4]. In recent years, before the COVID-19 pandemic, the annual number of TM visits has increased enormously, with an estimated compound annual growth rate of 52% from 2005 to 2014 and 261% from 2015 to 2017 [5]. The current health emergency is rapidly transforming the medical care system, driving the use of TM to a further exponential increase, the full extent of which is still being measured with the present knowledge [6]. Early estimates conducted by McKinsey & Company suggested that telehealth increased 38-fold during the timeframe winter 2020 and winter 2021, with a usage peak during April 2020 and subsequent stabilization in the subsequent months [6].

Telepsychiatry (TP) refers to the usage of ICTs in mental health care and treatment. It represents one of the earliest adaptations of TM in the field of medicine [7]. Nowadays, TP is the second most applicable type of TM globally (following teleradiology) [8,9]. If compared to TP, the concept of telemental health (TMH) has a more recent origin and a broader meaning, encompassing all technology-mediated modes of communication and referring to the use of ICTs for diagnostic, therapeutic, preventive, educational, and administrative purposes. The services supplied by TMH are provided not only by psychiatrists but also by a broader range of professionals. Further, TMH includes both synchronous (that take place simultaneously in different locations—such as videoconferencing or via telephone) and asynchronous modalities (that occur at different places and times—such as messaging or smartphone apps) [10]. Videoconferencing represents the most frequently applied modality, approaching the traditional setting of the doctor-patient interview [11]. The latest denomination of TMH is e-mental health (EMH), which similarly relates to the provision of care services through electronic media. EMH refers to a user-centered model of care, increasingly personalized to the user’s needs [12]. Another contemporary terminology is digital psychiatry (DP), defining a highly tailored and confidential care relationship through the use of an intuitive interface, and Digital Health Interventions (DHI) which include all health interventions virtually delivered [13].

The COVID-19 pandemic disproportionately impacted mental health services worldwide [14,15,16,17]. The pandemic-related outbreak and relative restrictive measures determined, on the one hand, the abrupt discontinuation in the traditional mental health care; on the other hand, the rapid modification of services to guarantee both the continuity of treatment in usual patients and the access of new ones [18,19,20]. Among these adaptations, the implementation of TMH and TP services has allowed the substitution of traditional in-person interventions in respect of the norms of physical distancing [21]. However, not all countries and mental health services, including Italy, were adequately prepared for this “digital revolution” since TP-related topics are rarely included in academic formation. Therefore, the primary objective of the present work aims to preliminarily explore the level of training, knowledge, beliefs, and experiences in the field of DP and related topics (i.e., TM, TP, TMH, EMH, DP, DHIs) at different stages of training in a cohort of Italian medical students, psychiatry trainees, and early career psychiatrists (ECPs). Secondly, explore if any determinants (i.e., the level of training, the level of clinical practice experience) may influence the level of knowledge of DP and related topics. Finally, explore if any determinants (i.e., the level of training, the level of clinical experience, the level of knowledge in DP and related topics) may influence the beliefs and/or opinions of Italian medical students, psychiatry trainees, and early career psychiatrists (ECPs) in the field of DP and related topics (i.e., TM, TP, TMH, EMH, DP, DHIs).

## 2. Materials and Methods

An ad hoc cross-sectional online survey was designed using Google LLC Forms and disseminated, in both digital and paper form, from 28 September 2020 to 7 April 2021. The link to participate was shared using social platforms, such as Facebook, WhatsApp, and Instagram. The survey was also administered in paper form to all medical students who attended the SOD Unit of Clinical Psychiatry at the Azienda Ospedaliero-Universitaria “Ospedali Riuniti”, in Ancona, Italy.

All recruited participants met the following inclusion criteria: (a) being an Italian medical student, a medical doctor waiting to start a psychiatry training program, a psychiatry trainee, or an ECP (i.e., a young psychiatrist within five years of their psychiatry training program or less than 40 years old); (b) being able and willing to provide consent to participate in the study and authorization to analyze data for research purposes; (c) filling out all sections and questions of the survey.

The survey consisted of four main sections. The first focused on sociodemographic data and included ten questions (seven multiple-choice and three open-ended questions). This first section was designed to collect general data on participants (i.e., sex, age, civil status, socio-economic status, current year of university study, current year of psychiatry training, how many years post-psychiatry training program were passed, country of origin, country of university studies, and so forth).

The second section consisted of 20 questions (14 multiple-choice and six 5-point Likert rating scale questions) designed to collect information regarding academic training (if any) in the field of DP and related topics (i.e., TM, TP, TMH, EMH, DP, DHIs). The six 5-point Likert rating scale items (items 9–14 of the second section) were developed to assess the level of participant’s perceived significance derived by the implementation of DP and related topics (i.e., TM, TP, TMH, EMH, DP, DHIs) during the Faculty of Medicine and during the Psychiatry Training Program, that it was named ‘Perceived Significance Index’ (PSI). The PSI is the sum of items 9–14 of the second section and ranges 5–25. The internal consistency was determined from Cronbach’s alpha calculation, considering that a Cronbach’s alpha of 0.70 or higher was adequate if the objective of the scale is for use in research [22].

The third section included 19 multiple-choice questions designed to explore participants’ level of knowledge on the topic. This section was used to build an index named Knowledge Score (KS), indicating the overall level of knowledge of the participants based on the sum of their correct answers. The KS represents a continuous variable that was developed to compare the level of knowledge in the field of DP and related topics according the level of training (i.e., the four subgroups constituted by medical students, medical doctors waiting to start a psychiatry training program, psychiatry trainees and ECPs), and according to the level of clinical experiences in the field of DP and related topics (i.e., TM, TP, TMH, EMH, DP, DHIs), as measured by the exploratory items contained in the second section specifically investigating which topics of DP and at which levels of training have been taught.

The fourth section consisted of 34 questions (5-point Likert rating scale) designed to investigate participants’ opinions on DP and related topics (i.e., TM, TP, TMH, EMH, DP, DHIs). The total score derived by the sum of all items of the fourth section was named Digital Psychiatry Opinion index (DPO). The Kaiser-Mayer-Olkin (KMO) measure of sampling adequacy suggested that the sample was favourable (KMO = 0.936) and the Bartlett’s test of Sphericity was highly significant (χ^2^ = 6723.041, df = 561, *p* < 0.001). Thereafter, an exploratory factor analysis (EFA) was performed with the principal components extraction method and the Kaiser-Varimax rotation method. The number of factors was determined by the size of eigenvalues (>1) and the variance explained by each factor, as well as the coherence and interpretability of the factors. Items allocated to a specific factor were based on a loading of more than 0.50 on the corresponding factor, and items were excluded when the difference of factor loadings was less than 0.49. Lastly, the internal consistency was determined from Cronbach’s alpha calculation, considering that a Cronbach’s alpha of 0.70 or higher was adequate [22].

Statistical analysis, including EFA, was conducted using Statistical Package for Social Sciences (SPSS) software for macOS (version 26.0, IBM Corp., Armonk, NY, USA, 2019). Categorical variables were summarized as frequencies and percentages (N; %), while continuous variables (age, PSI, KS, DPO and related factors) were reported as mean and standard deviation (S.D.). The normality of continuous variables was analyzed by using the Kolmogorov-Smirnov and Shapiro-Wilk normality tests. Student’s *t*-test or analysis of variance (ANOVA), when appropriate, were used to compare the KS according to: (1) the level of training (i.e., the four subgroups constituted by medical students, a medical doctor waiting to start a psychiatry training program, psychiatry trainees and early career psychiatrists; (2) the level of theoretical and/or practical training experiences (i.e., whether and which DP and related topics were taught from a theoretical and/or practical point of view). Mann-Whitney U or Kruskal-Wallis test, when appropriate, were used to compare the PSI, DPO and related factors according to: (1) the level of training (i.e., the four subgroups constituted by medical students, a medical doctor waiting to start a psychiatry training program, psychiatry trainees and early career psychiatrists; (2) the level of theoretical and practical training experiences (i.e., whether and which DP and related topics were taught from a theoretical and/or practical point of view). Bivariate Pearson’s correlations were used to investigate potential relationships between DPO and KS, DPO and PSI, and KS and PSI scores. Linear regression analysis was performed to investigate the associations between DPO and KS, DPO and PSI, and KS and PSI scores. The level of significance was set at α ≤ 0.05, and all hypotheses were two-tailed.

## 3. Results

### 3.1. Sociodemographic Results

Key sociodemographic characteristics are summarized in Table 1. The final sample included 239 subjects, of which the majority were female. The mean age was 26.6 (±SD = 3.9) years, ranging from 19 to 41 years. Regarding marital and economic status, the majority were single/unmarried and belonging to the middle class. Regarding current college/work status, slightly over a half were medical students, and the remaining were newly qualified doctors, psychiatry trainees, and ECPs, approximately one-third each one. Among medical students, the majority attended the fifth or sixth year. Within the sample of psychiatry trainees, most reported attending the first year of their psychiatry training program. The majority of the sample stated that they were studying/working in Italy.

### 3.2. Training in Digital Psychiatry

Most of the sample stated that the topics of TM, EH, EMH, and DP had not been taught within their medical school training, and, whereas these topics were discussed in the academic setting, the time dedicated to them was little, i.e., less than 20% of the total training time (N = 222; 92.9%). Nevertheless, more than half of the sample declared that implementing a course/module about TM (N = 133; 55.6%) or EH (N = 148; 62%) within the medical school would be important. Similarly, almost all the sample stated that no training in DP was provided within the psychiatry training program or they were unaware of it (N = 230; 96.2%). However, the majority declared that implementing a course/module on TP (N = 174; 72.8%), DP (N = 182; 76.2%), or EMH (N = 185; 77.4%) within the psychiatry training program would be important. Furthermore, 33 subjects reported to have applied their DP-related knowledge in their clinical practice (13.8%), and 11 declared to have used it moderately (i.e., about 1–2 times/month) even before the onset of the COVID-19 pandemic (4.6%). Finally, 15 subjects reported that they had never applied DP before the COVID-19 pandemic (6.3%), and 11 declared that the pandemic slightly intensified the use of DP (4.6%). The PSI score showed an excellent internal consistency (Cronbach’s a of 0.92). The mean average of PSI was 23.1 (SD = 4.7), without any significant sex-based differences (*p* = 0.496) (Table 2). Interestingly, psychiatry trainees showed significantly lower PSI scores compared to ECPs (*p* = 0.007) and newly qualified M.D. waiting for a psychiatry training program (*p* = 0.011); while medical students showed significantly lower PSI scores compared to ECPs (*p* = 0.047) (Table 3).

### 3.3. Level of Knowledge on Digital Psychiatry

More than half of the sample (N = 134; 56.1%) gave an incorrect definition of EH; conversely, most of the sample correctly defined TP (N = 176; 73.6%) and TM (N = 137; 57.3%). Regarding the main targets of TM, the majority of the sample indicated most of the correct answers. Concerning the sub-specialties included in the DP, over half of the sample correctly identified TP, EMH, and TM, while only the minority of the sample indicated phone-, chat-, and email-based psychiatric counseling, smartphone apps, social media. Regarding DHIs, only 24.3% (N = 58) of the participants correctly replied that they require neither physical nor temporal co-presence of clinician and patient. There were conflicting responses regarding which platforms/tools could be included in DHIs.

Obsessive-compulsive disorder (OCD), post-traumatic stress disorder (PTSD), and phobias were correctly identified by the majority of the sample (N = 175; 73.2%) as mental health conditions that may be treated by using digital interventions. The majority of the sample (N = 136; 56.9%) correctly identified PTSD and attention deficit hyperactivity disorder (ADHD) as conditions for which TP is recommended. The majority of the sample (N = 164; 68.6%) also indicated paranoia and the paranoid state as contraindicated conditions in TP, in line with the International Psychoanalytic Association (IPA) guidelines. Conversely, only 22.2% of the sample (N = 53) indicated severe trauma as contraindicated conditions in TP.

Almost all the sample could not determine the historical origin of TP, while most of the sample recognized the country in which TP was born and identified videoconferencing as the most commonly used mode of communication in TP; only one-fifth of the participants were able to indicate Zoom as the platform recommended by the European Association for Psychotherapy (EAP) guidelines.

According to most participants, the patient should be able to access the Internet and use the electronic device independently (N = 210; 87.9%) and has to be skilled in electronic device characteristics and the management of their sensitive data (privacy and confidentiality) (N = 204; 85.4%). Most of the sample believe that the mental health professional should investigate the patient’s attitude towards online treatment (N = 177; 74.1%) and obtain informed consent (written form) before providing a TP consultation (N = 172; 72%). In contrast, over half of the sample erroneously declared that the clinician should attend a training course/obtain a certification to use DP (N = 141; 59%).

The mean average KS was 9.9 (SD = 2.7), with statistically significantly higher scores in women (*p* = 0.023), psychiatry trainees and ECPs (in both cases, *p* < 0.001) (Table 4). As expected, statistically higher KS were reported among those who gave a correct definition of DP (*p* = 0.01), TP (*p* = 0.02), and DHIs (*p* < 0.001). Time dedicated to the topic during the training did not influence KS, even though a trend was observed (*p* = 0.07). Higher KS scores were observed in those who had applied their knowledge in digitally-delivered mental health interventions, acquired during their university and post-lauream studies, into their clinical practice (*p* < 0.001), had applied digital interventions moderately even before the COVID-19 pandemic (*p* = 0.04), had moderately or substantially increased the frequency in their use of DP during the pandemic (*p* = 0.03) (Table 4).

### 3.4. The Digital Psychiatry Opinion

The majority of the sample declared that TM might improve healthcare in various conditions. Responses were uneven regarding DP’s potential to provide interventions comparable to those in-person and ensure adequate privacy. More than half of the sample agreed (N = 134; 56.1%) that DP did not affect the building of a good therapeutic alliance with the patient. However, most of the sample stated that, before providing DP interventions, the clinician should accurately assess the risks versus benefits of the digital tool (N = 200; 83.7%). They also declared that DP should mainly be used for follow-up visits of already known and stable patients (N = 192; 80.4%) and that it should not be recommended during a first assessment visit (N = 179; 74.9%).

Overall, most participants declared that DP cannot wholly replace traditional in-person interventions (N = 170; 71.2%). Moreover, more than half of the sample believe that DP synchronous interventions are more effective than the asynchronous ones (N = 151; 63.1%), and a substantial part of the sample declared that DP should be provided just in synchronous mode (N = 100; 41.9%). Only 28.4% of the sample (N = 68) correctly declared that digital interventions are effective as in-person interventions, being significantly reported among those participants who declared to have received training in DP (*p* = 0.01).

EFA and scree plot indicated that 30 items out of the initial 34 items of the fourth section, loaded onto five factors with eigenvalues greater than one should be retained, accounting for 67.6% of the total variance (Figure S1). Factor 1 (named ‘Mental Health Services Improvement of Digital Interventions’, F1) consisted of 10 items (range 10–50) explaining 45.6% of the total variance. Factor 2 (named, ‘Preliminary needed technical requirements and indications for delivering digital mental health interventions’, F2) consisted of six items (range 6–30) and accounted for 9.3% of the total variance. Factor 3 (named, ‘Basic needed training requirements for delivering digitally-mediated mental health interventions’, F3) consisted of six items (range 6–30) accounting for 5.6% of the total variance. Factor 4 (named, ‘Opinions regarding the comparable efficacy between in-person versus digitally-delivered mental health interventions’, F4) consisted of four items (range 4–20) and explained 3.8% of the total variance. Factor 5 (named, ‘Usability of digitally-delivered mental health interventions in special/critical situations’, F5) consisted of four items (range 4–20) and accounted for 3.3% of the total variance. Pearson’s correlations analyses showed that there were significant positive correlations among these five factors. Analysis of the internal consistency showed an excellent internal reliability in the DPO index (Cronbach’s α of 0.961), and excellent reliability in the following retained factors (Cronbach’s α of 0.945 for F1, Cronbach’s α of 0.903 for F3, Cronbach’s α of 0.910 for F5. While a good internal consistency was shown for F2 (Cronbach’s α of 0.887) and acceptable for F4 (Cronbach’s α of 0.786).

The mean average of DPO was 111.40 (SD = 19.2), without any significant sex-based differences (*p* = 0.414), neither for the level of training (*p* = 0.373), the type of training provided during medical school and/or psychiatry training programs (*p* = 0.118), clinical practice experience before the COVID-19 pandemic (*p* = 0.376) and after COVID-19 pandemic (*p* = 0.658). The mean average of F1 was 38.4 (SD = 8.1), without any significant differences for sex (*p* = 0.392), for level of training (*p* = 0.261), for the presence/absence of TM (*p* = 0.699), EH (*p* = 0.378), EMH (*p* = 0.937), DP training (*p* = 0.533). No significant differences were found depending on the type of training acquired in DP (theoretical/practical) (*p* = 0.410) or the level of clinical practice in DP (*p* = 0.647).

The mean average of F2 was 23.7 (SD = 4.5), without any significant differences for sex (*p* = 0.199), for level of training (*p* = 0.831), for the presence/absence of TM (*p* = 0.537), EH (*p* = 0.373), EMH (*p* = 0.263), DP training (*p* = 0.155). No significant differences were found depending on the type of training acquired in DP (theoretical/practical) (*p* = 0.424) or the level of clinical practice in DP (*p* = 0.239).

The mean average of F3 was 22.6 (SD = 4.5), without any significant differences for sex (*p* = 0.730), for level of training (*p* = 0.768), for the presence/absence of TM (*p* = 0.378), EH (*p* = 0.131), except for EMH (*p* = 0.019) and DP training (*p* = 0.010). No significant differences were found depending on the type of training acquired in DP (theoretical/practical) (*p* = 0.109) or the level of clinical practice in DP (*p* = 0.601).

The mean average of F4 was 10.5 (SD = 3.2), without any significant differences for sex (*p* = 0.571), for level of training (*p* = 0.336), for the presence/absence of TM (*p* = 0.935), EH (*p* = 0.354) and DP training (*p* = 0.230), except for EMH (*p* = 0.027). No significant differences were found depending on the type of training acquired in DP (theoretical/practical) (*p* = 0.118) or the level of clinical practice in DP (*p* = 0.185).

The mean average of F5 was 16.3 (SD = 3.3), without any significant differences for sex (*p* = 0.534), for level of training (*p* = 0.259), for the presence/absence of TM (*p* = 0.592), EMH (*p* = 0.109) training, except for EH (*p* = 0.015) and DP training (*p* = 0.046). F5 scores were significantly higher in psychiatry trainees belonging to the first year of their psychiatry training program, compared to senior psychiatry trainees (*p* = 0.007) and in those who declared to have already used DP before the COVID-19 pandemic (*p* = 0.011). No significant differences were found depending on the type of training acquired in DP (theoretical/practical) (*p* = 0.666) or the level of clinical practice in DP (*p* = 0.370).

### 3.5. Associations between KS, PSI and DPO

**Figure 1:**
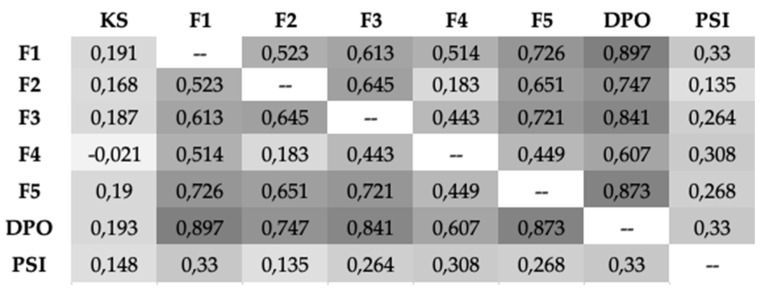
Pearson’s Correlations’ Heatmap. KS: Knowledge Score; DPO: Digital Psychiatry Opinion; PSI: Perceived Significance Index; F1: factor 1; F2: factor 2; F3: factor 3; F4: factor 4; F5: factor 5. Higher correlations are marked with darker shades, while lower correlations are marked with lighter shade.

**Figure 2:**
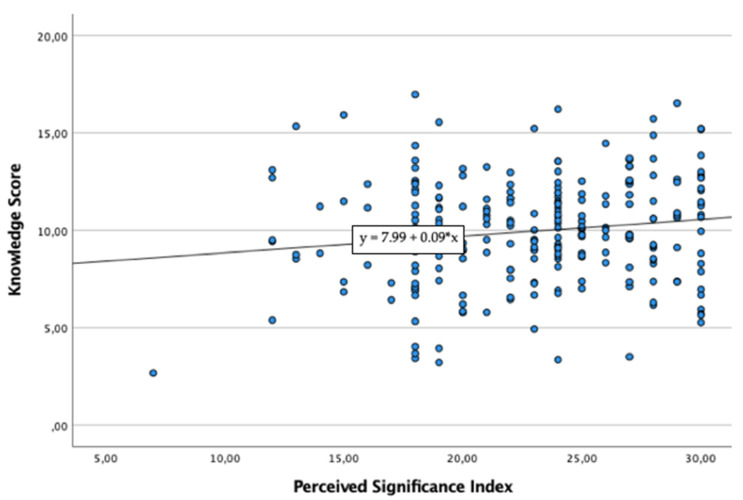
Linear Regression Model between Knowledge Score (dependent variable) and Perceived Significance Index (independent variable/predictor).

**Figure 3:**
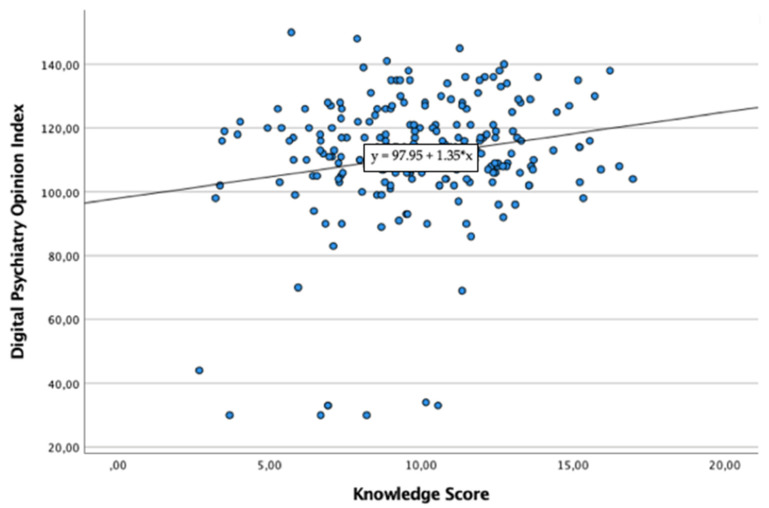
Linear Regression Model between Digital Psychiatry Opinion Index (dependent variable) and the Knowledge Score (independent variable/predictor).

Bivariate correlations analyses demonstrated strong positive significant correlations between KS and PSI (r = 0.148, *p* < 0.001), KS and DPO (r = 0.193, *p* < 0.001) and KS and each factor of DPO (Figure 1). Linear regression analysis demonstrated that PSI scores statistically significantly predicted KS total scores (F(1, 237) = 5.283, R^2^ = 0.022, *p* = 0.022) (Figure 2). Linear regression analysis demonstrated that KS scores statistically significantly predicted DPO total scores (F(1, 237) = 9.136, R^2^ = 0.037, *p* = 0.003) (Figure 3).

## 4. Discussion

At the time of writing, this study represents the first Italian one to investigate the level of training and attitudes of medical students and young mental health professionals in the field of DP. Despite their long history, DP-related disciplines appear to be poorly addressed within the Italian academic formation, leading to a lack of knowledge on the topic. Although some participants possess general notions regarding TM and TP, specialty and detailed knowledge are often lacking. In addition, only a limited number of subjects demonstrated awareness about the recent introduction of digital tools in clinical practice. Nevertheless, most participants expressed interest in having these topics addressed during their training, particularly ECPs and newly qualified medical doctors waiting to start a psychiatry training program. The reason would be mainly explained by the same starting working condition of both categories who are going to start working on the frontline and may feel the need to possess more technical skills also in the field of DP, due to the current COVID-19 pandemic. Moreover, early career (and inexperienced) health professionals are those more prone to consider DP and related disciplines significantly important to be implemented in medicine courses and/or psychiatry training programmes, being those who showed higher PSI scores, compared to more senior (mental) health professionals. PSI significantly predicts KS, probably due to the fact that a positive and propositive attitude towards DP and related disciplines can predispose mental health professionals to deepen this topic, also independently by a formal academic DP training. In particular, our findings demonstrated that to be female and trained in DP-related topics is significantly associated with higher KS. Significantly higher KS scores were also found among ECPs and psychiatry trainees and this could reflect the initial hypothesis that at a later stage of psychiatry training each participant could have received a formal (practical and/or theoretical) DP training. Interestingly, the most significantly higher KS scores were found among those who declared to have received a practical (formally and/or informally) clinical experience in delivering digital mental health interventions. Furthermore, KS significantly predicts DPO, by underlining how knowing and to be informed about DP can significantly influence participant’s DP opinion and attitude and, hence, their application of DP interventions in their routinely clinical practice. In fact, those participants who declared to be trained in DP and EMH showed significantly positive opinions regarding which professional and training skills are needed to provide digital mental health interventions (factor 3), think that in-person versus remote digital mental health interventions are comparable in terms of efficacy (factor 4) and that digital mental health interventions may represent a useful tool in those critical/emergency situation, such as the current COVID-19 pandemic (factor 5). Indeed, the current COVID-19 pandemic may have influenced our findings as the study was carried out during the second Italian COVID-19 pandemic wave, as demonstrated by the highest opinions towards the usability of digital mental health interventions during crisis/emergency situations provided by psychiatry trainees at their early stage.

Overall, the present findings of the inadequate DP training in Italy are in line with data collected in other European countries, such as France [23], and in non-European countries, such as India [24], Sri Lanka [25] and Asia-Pacific Regions [26]. Similarly, the overall interest in implementing the field of DP is consistent with past findings in samples of medical students and psychiatry trainees [24,25,26,27,28,29,30,31]. Conversely, different data emerge from U.S. studies conducted in the last decade. According to these, one-fifth of respondents had received formal training in TP already during medical school [27] and almost half of them during psychiatry training programs [32].

Most participants shared the same beliefs regarding both advantages and limitations of DP. The large majority declared that the application of ICTs could facilitate treatment access and continuity in case of reduced mobility, geographic isolation, or health emergencies. Moreover, most participants believe that digitalization could result in cost-saving, bringing a concrete benefit to the National Health Service, and a better rationalization of social and health care processes. These findings are in accordance with those emerging from previous studies [15,29,33,34].

At the same time, in line with earlier findings [35,36], data showed a general hesitation and lack of confidence regarding digital interventions. In particular, the perplexities concerned the effectiveness of digital interventions compared to in-person ones, despite the evidence proving that the two modes are equally effective in terms of outcomes, treatment adherence, and symptomatology improvement [37]. Only a tiny percentage of the sample declared that digital interventions are as effective as in-person ones, following data emerging from existing literature [30,38,39].

In this regard, the present work showed that being trained in DP is significantly associated with the belief that DP is equally effective to in-person care. Indeed, the knowledge acquired through adequate (mainly practical) training on DP can favorably influence the perception of its usefulness and validity in clinical practice [40]. It therefore seems undeniable that the lack of education and clinical practice in this field, leading to a negative influence on clinicians’ opinions, is a critical limiting factor in the process of psychiatry digitization [36,41].

Moreover, data revealed a general concern about achieving a good therapeutic alliance through digital tools. These results are consistent with existing literature [11,30,34,35,39], and in contrast with the evidence that patients treated via videoconferencing tend to consider the therapeutic alliance valid [42]. Other perplexities were related to data and privacy protection and the appropriateness of using DP during the first psychiatric visit rather than just in follow-up visits in already stable patients. Likewise, those concerns are in line with previous research [34,35,40].

These preliminary findings should also be considered in light of the limitations that this first study presents. First, the choice of a mixed-mode data collection (both hand-to-hand and digital form). In fact, the digital form did not allow to verify the actual occupational/work belonging and, hence, to ensure the respect of inclusion criteria. In addition, the digital modality does not prevent filling out the questionnaire more than once. Second, the study’s cross-sectional nature allowed us to have the current snapshot of the Italian situation but did not provide an observation over time (i.e., comparison between before and after academic training). Third, the period of data collection during the COVID-19 pandemic may have determined an overestimation of the KS and level of interest/attitude towards DP (i.e., the recent rapid spread of DP could have impacted KS). Fourth, the small sample size may have undermined the sample’s representativeness, which may vary in its characteristics on a regional basis. Finally, the sample of medical students and psychiatry trainees was widely more represented than ECPs. This may explain the low values of KS and the poor training on the issue. Similarly, among psychiatry trainees, most of the sample was represented by those attending the first year, i.e., at the very beginning of their training.

Therefore, our findings may interestingly address more specific changes in DP training, as they underline how it is significant to implement education and clinical practice in DP and related disciplines since the course of medicine and then during psychiatry training programmes. Another important point regards the presence of a mentorship program within psychiatry training programmes which could significantly improve the PSI as well as DPO among early career (mental) health professionals, as it has been demonstrated that own a positive attitude towards the importance to implement DP and related disciplines in university and post-university courses may significantly improve the level of knowledge and, then, the overall opinion in digital mental health interventions and, hence, incentivize their use in the routinary clinical practice. The current COVID-19 pandemic taught that digitalization is an essential part of the (mental) health services and infrastructures which should be greatly implemented as it may ensure the continuity to access and care in (mental) health services that could be abruptly discontinued due to the crisis/emergency situation and represent a protective factor towards the recrudescence of mental health conditions. Further studies with a greater sample size and more homogeneous geographic representation are needed to confirm what emerged from this research. Moreover, further longitudinal studies should appropriately stratify the results, considering the different academic and occupational categories and the variations in participants’ KS and attitudes over time.

## 5. Conclusions

In conclusion, the present study demonstrated the lack of formal training in DP within medical school and psychiatry training programmes, despite the recent digitalization of several health care services and medical specialties. The lack of theoretical and practical education during the academic years may represent a limiting factor to the spread of DP, resulting in a vast digitalization gap in today’s clinical practice. These findings reported that those who had received academic training had a more favorable opinion about digital mental health interventions and were more inclined to use them in their clinical practice. Overall, developing a toolkit of core competencies in the field of DP and including it within the formal training should be encouraged. Starting from a good education, we may see impressive increases in the digitization of psychiatry in the coming years.

## Figures and Tables

**Table 1:** Socio-demographic data of the sample.

Socio-Demographic Data of the Sample			
Variable	Answer	N	%
Sex	Male	92	38.5
	Female	147	61.5
Marital status	Single/unmarried	202	84.5
	Married/cohabiting	37	15.5
Economic status (optional item)	Low	15	6.3
	Average	175	73.2
	High	41	17.2
	Not known	8	3.3
Current position	Medical students	140	58.6
	*First year*	1	0.7
	*Second year*	1	0.7
	*Third year*	5	3.6
	*Fourth year*	10	7.1
	*Fifth year*	25	17.9
	*Sixth year*	98	70
	Newly qualified doctors	34	14.2
	Psychiatry residents	39	16.3
	*First year*	22	56.4
	*Second year*	7	18
	*Third year*	8	20.5
	*Fourth year*	2	5.1
	ECPs	26	10.9
	<*1 year*	8	30.8
	*1*–*2 years*	4	15.4
	*2*–*3 years*	2	7.6
	*4*–*5 years*	4	15.4
	*3*–*4 years*	8	30.8
Medical school country	Italy	236	98.7
	Foreign	3	1.3
Psychiatry residency country	Italy	63	96.9
	Foreign	2	3.1

N: frequency; %: percentage; ECP: Early Career Psychiatrist.

**Table 2:** Perceived Significance Index according to socio-demographic and training variables.

	Mean	SD	*p*-Value *^,^**
**Male**	22.8	4.8	* 0.496
**Female**	23.2	4.7	
**Single/unmarried**	22.9	4.7	* 0.150
**Married/cohabiting**	24	5	
**Low income**	22.9	4.8	** 0.680
**Medium income**	23.2	4.7	
**High income**	22.8	5.2	
**Medical Students**	22.8	4.8	** **0.015**
**Newly qualified M.D.**	24.6	3.6	
**Psychiatry residents**	21.7	4.4	
**ECP**	24.7	5.3	
**Medical Students**			** 0.558
*First Year*	n.m.	n.m.	
*Second Year*	n.m.	n.m.	
*Third Year*	21.6	5.4	
*Fourth Year*	24.6	3.4	
*Fifth Year*	21.6	5.1	
*Sixth Year*	22.9	4.8	
**Psychiatry residents**			** 0.968
*First Year*	22	4.1	
*Second Year*	20.6	5.7	
*Third Year*	21.6	4.5	
*Fourth Year*	23	7.1	
**ECP**			** 0.575
*<1 year*	23.5	5.8	
*1–2 years*	22	6.7	
*2–3 years*	22	7	
*3–4 years*	26.2	5	
*4–5 years*	26.5	4.2	
**Taught TM in Medicine Faculty**			* 0.446
*Yes*	22.6	4.4	
*No*	23.1	4.8	
**Taught EH in Medicine Faculty**			* **0.025**
*Yes*	20.4	4.5	
*No*	23.2	4.7	
**Taught EMH in Psychiatry Training Programme**			* 0.308
*Yes*	20.4	4.7	
*No*	23.2	4.7	
**Taught DP in Psychiatry Training Programme**			** 0.882
*Yes*	21.8	5.7	
*No*	23.2	4.7	
**Type of DP Training**			** 0.151
*Only theoretical*	18	0	
*Only Practical*	n.m.	n.m.	
*Theoretical and Practical*	23.2	3.6	
**Clinical Practice in DP**			** 0.235
*Qualified, Yes*	24.3	4.7	
*Qualified, No*	23	4.7	
*Not Qualified, No*	22.8	4.8	
**How much COVID-19 pandemic favoured the implementation of DP in my clinical practice?**			** 0.324
*None change*	23.8	4	
*Slight change*	21.7	6.7	
*Moderate change*	26.3	3.7	
*Substantial change*	25.6	4	

M: mean; SD: standard deviation; ECP: Early Career Psychiatrists; n.m.: not measurable. * Mann Whitney’s U-test; ** Kruskal-Wallis test. Bold number indicates significant *p*-value.

**Table 3:** Pairwise comparisons PSI according to the level of training.

Sample 1–Sample 2	Statistics of Test	Standard Error	Statistics of Standard Test	*p*-Value
Psychiatry Trainee vs. Medical Student	18.017	12.472	1.445	0.149
Psychiatry Trainee vs. Newly qualified medical doctor	41.127	16.162	2.545	**0.011**
Psychiatry Trainee vs. ECP	−47.192	17.440	−2.706	**0.007**
Medical Student vs. Newly qualified medical doctor	−23.110	13.170	−1.755	0.079
Medical Student vs. ECP	−29.175	14.710	−1.983	**0.047**
Newly qualified medical doctor vs. ECP	−6.066	17.946	−0.338	0.735

ECP: early career psychiatrist. Each line runs a statistical test according to the null hypothesis that the distribution between sample 1 and sample 2 are identical. The asymptotic significance (2-way) are represented in the table with a significance level set at 0.05. Bold number indicates significant *p*-value.

**Table 4:** Knowledge Score according to socio-demographic and training variables.

	Mean KS	SD	Statistical Test *^,^**	*p*-Value
**Male**	9.5	2.5	* t(237) = −2.281	**0.023**
**Female**	10.3	2.8		
**Single/unmarried**	9.7	2.7	** F(1) = 12.403	**0.001**
**Married/cohabiting**	11.4	2.7		
**Low income**	9.9	2	** F(3) = 0.377	0.77
**Medium income**	9.9	2.8		
**High income**	10.3	2.5		
**Medical Students**	9.1	2.4	** F(3) = 15.046	**<0.001**
**Newly qualified M.D.**	10.4	2.9		
**Psychiatry residents**	11.2	2.2		
**ECP**	12.1	2.8		
**Medical Students**			** F(5) = 0.492	0.782
*First Year*	10.2	n.m.		
*Second Year*	9.3	n.m.		
*Third Year*	7.8	2.6		
*Fourth Year*	9.1	2.7		
*Fifth Year*	8.7	2		
*Sixth Year*	9.2	2.6		
**Psychiatry residents**			** F(3) = 0.176	0.912
*First Year*	11.1	2.4		
*Second Year*	10.4	2.1		
*Third Year*	11.3	3.6		
*Fourth Year*	10.8	2.2		
**ECP**			** F(4) = 1.407	0.265
<*1 year*	10.8	3.2		
*1*–*2 years*	12.7	1.8		
*2*–*3 years*	9.5	5		
*4*–*5 years*	11	0.5		
*3*–*4 years*	13.5	3.3		
**Taught TM in Medicine Faculty**			* t(237) = −0.570	0.569
*Yes*	9.7	2.3		
*No*	10	2.8		
**Taught EH in Medicine Faculty**			* t(237) = −0.848	0.397
*Yes*	9.3	3.5		
*No*	10	2.7		
**Taught EMH in Psychiatry Training Programme**			* t(213) = −1.177	0.24
*Yes*	8.9	2.7		
*No*	10.1	2.7		
**Taught DP in Psychiatry Training Programme**			* t(216) = −1.786	0.075
*Yes*	8	2.8		
*No*	10.2	2.7		
**Type of DP Training**			** F(2) = 0.977	0.429
*Only theoretical*	2.6	4.2		
*Only Practical*	11.7	n.m.		
*Theoretical and Practical*	11.7	4.7		
**Clinical Practice in DP**			** F(2) = 8.827	**<0.001**
*Qualified, Yes*	11.2	3		
*Qualified, No*	10.5	2.9		
*Not Qualified, No*	9.3	2.4		
**How much COVID-19 pandemic favoured the implementation of DP in my clinical practice?**			** F(3) = 3.327	**0.033**
*None change*	9.5	3.7		
*Slight change*	10.6	1.8		
*Moderate change*	13.2	1.5		
*Substantial change*	12.2	2.8		

M: mean; SD: standard deviation; ECP: Early Career Psychiatrists; n.m.: not measurable. * Student’s *t*-test; ** ANOVA. Bold number indicates significant *p*-value.

## Data Availability

Data supporting reported results are available, upon request to corresponding author.

## Supplementary Materials

The following supporting information can be downloaded at: https://www.mdpi.com/article/10.3390/healthcare10020390/s1, Figure S1: Scree Plot.
