# Supplementary figures and images for: A Preliminary Italian Cross-Sectional Study on the Level of Digital Psychiatry Training, Knowledge, Beliefs and Experiences among Medical Students, Psychiatry Trainees and Professionals
Source: Healthcare (Basel). 2022 Feb 18;10(2):390. doi: 10.3390/healthcare10020390 (PMC8871870; doi:10.3390/healthcare10020390)

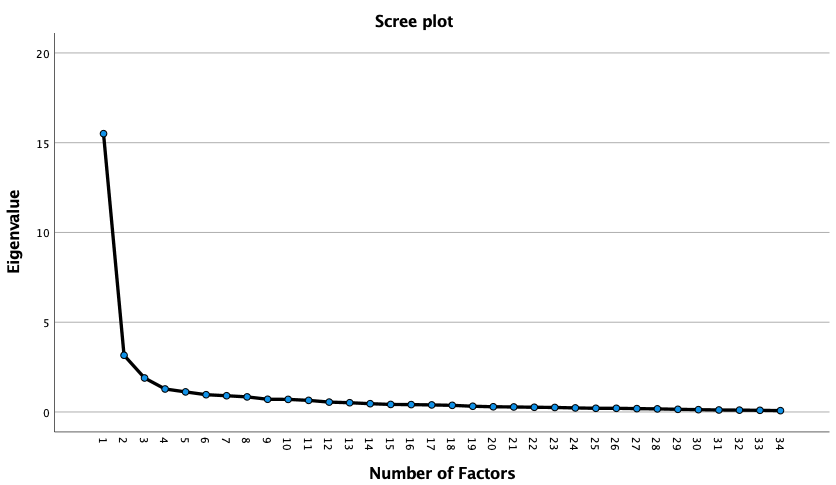

Supplement: Supplementary file 1 [file healthcare-10-00390-s001.zip › healthcare-1532339-supplementary Figure S1.png]
